# Production of Basil and Eucalyptus Seedlings in Substrates Amended with Biochars Derived from Municipal Solid Waste and Lemongrass Biomass

**DOI:** 10.1021/acsomega.6c02939

**Published:** 2026-07-14

**Authors:** Vanessa Susana Rech Bisi, Wendel Paulo Silvestre, Marcelo Godinho, Gabriel Fernandes Pauletti

**Affiliations:** 58802 University of Caxias do Sul, Postgraduate Program in Process Engineering and Technologies, Caxias do Sul 95070560, RS, Brazil

## Abstract

The reuse of municipal solid waste and agro-industrial biomass has been considered a potential alternative to promote environmental sustainability and the circular economy. In this context, the present study evaluated the agronomic potential of biochars produced from municipal solid waste (BMSW) and lemongrass biomass (BCL), incorporated at different concentrations (5.0, 7.5, and 10.0 wt %) into a commercial substrate to produce basil (*Ocimum basilicum*) and eucalyptus (*Eucalyptus grandis*) seedlings. Biometric parameters and nutritional content of the plants were evaluated. The biochars were characterized by Fourier transform infrared spectroscopy (FTIR) and X-ray diffraction (XRD) to identify surface functional groups and mineral phases. The analyses indicated differences in the chemical and structural composition of the materials. The results demonstrated that the responses varied according to the plant species and the biochar source. BCL was associated with a greater biometric development of basil seedlings, especially in root growth, while BMSW was linked to a greater development of the aerial part of eucalyptus seedlings, mainly at concentrations of 5.0 and 7.5 wt %. In both species, the incorporation of biochars possibly yielded the accumulation of dry biomass. Regarding nutrients, potassium content was higher in treatments with 7.5 wt % BCL, reaching 15.3 g kg^–1^ in basil and 24.3 g·kg^–1^ in eucalyptus. For eucalyptus, the highest calcium levels were observed in treatments with BMSW, while higher boron levels were found in basil grown with 10.0 wt % of this biochar. Overall, the results indicate that the agronomic performance of biochars depends on their physicochemical properties and the interaction with the specific requirements of each plant species. However, the interpretations related to the action mechanisms must be considered cautiously since pH, electrical conductivity, porosity, water retention capacity, nutrient availability, and other physical-chemical Properties of the substrate blends after biochar incorporation were not measured. Thus, the results represent preliminary evidence obtained under controlled greenhouse conditions, highlighting the potential of biochars derived from waste as substrate conditioners in seedling production.

## Graphical abstract

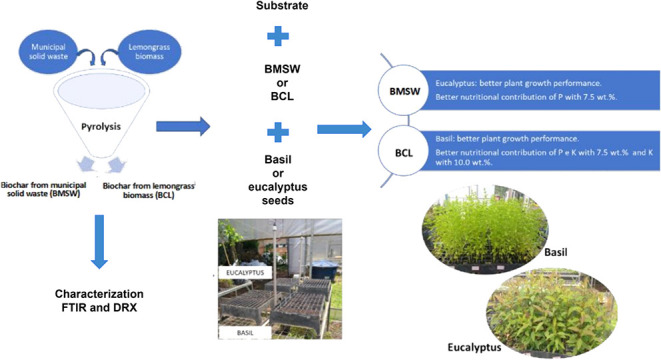

## 1 Introduction

Sustainable development has become a global priority in response to increasing environmental pressures and the need to use resources more efficiently. To this end, the UN established the 2030 Agenda with 17 Sustainable Development Goals (SDGs), aiming, among other things, to protect the planet’s environment and climate.^1^ Therefore, there is a growing demand for alternatives that mitigate soil degradation while addressing climate change and the pressure to expand agricultural areas through waste reuse.

According to data from the International Bank for Reconstruction and Development, 2.01 billion tons of municipal solid waste (MSW) are generated annually, 33% of which are managed in an environmentally inappropriate way.^2^ By 2050, 3.4 billion tons of MSW will be generated each year. In 2022, Brazil generated 81.8 million tons of MSW, representing 224.1 thousand tons per day and, on average, 1.043 kg of MSW per inhabitant per day.^3^ For the disposal of MSW, selective collection is present in only 75.1% of all municipalities in the country,^3^ and landfilling MSW has become a complex and increasingly costly action for public authorities due to the need to obtain suitable areas for the installation of these landfills, as well as the implementation of the necessary infrastructure to resolve environmental problems with contaminants in the soil and surface and underground waters, disease vectors and emission of toxic and/or harmful gases to the environment.^4^

Regarding agricultural activity, it aims to produce food, fibers, and raw materials to obtain bioproducts, bioinputs, and bioenergy. Agriculture and agroindustry are characterized by activities across different segments that use and generate biomass from agricultural, livestock, or forestry raw materials.^5^ The agro-industrial waste produced consists of stems, leaves, roots, fruit peels, and seeds, which are customarily discarded during production, postharvest, and processing.^6^ The biomass derived from these wastes exhibits peculiarities in composition, seasonality, heterogeneity, and perishability.^5^ Scientific and technological advances that enable the development of new plant materials, such as genetic improvement, which increases biomass, and new industrial processes for processing plant-derived products, result in the generation of large quantities of residual plant biomass.^5^ The annual global generation of dry waste from agricultural and forestry practices is estimated at 140 Gt, with Brazil responsible for approximately 451 Mt; cereal production alone accounts for 66% (298 Mt) of this waste.^6^ The reuse of agro-industrial waste is guided by the principles of bioeconomy and circular economy, to promote sustainable value chains based on renewable resources. The result is the production of bioproducts derived from biomass that can be used in various areas, such as agriculture.^5^

The growth in agricultural demand and agroindustries tends to increase the quantity and heterogeneity of available biomass. The essential oil (EO) industry sold 253,000 t on the international market in 2021, with the prospect of increasing to 345,000 t by 2026, driven mainly by the phytotherapeutic effects of EOs and their desirable characteristics for perfumery and cosmetics.^7^

Among the OE exported are citrus oils (orange, bergamot, petitgrain), mints (mint, *Mentha piperita*), palm rose, eucalyptus, citronella, and lemongrass, which present characteristics of interest to the national and international market, mainly considering purified substances such as limonene, citral, citronellal, eugenol, menthol and safrole, which are used for specific purposes.^7^

In the case of lemongrass (*Cymbopogon citratus*) EO, it is mainly composed of the monoterpene citral (approximately 60 wt %), consisting of the isomers citral A (geranial) and citral B (neral), with the monoterpene myrcene also present with a content of 15–40 wt %. These compounds are responsible for EO’s fungicidal, bactericidal, and insecticidal action, resulting in high demand for this product.^8,9^ The extraction of EO generates a high amount of lemongrass biomass since it is necessary to produce 60–90 t·ha^–1^year^–1^ of leaves to produce 160–220 kg·year^–1^ of EO, as estimated for São Paulo state.^10^

To sustainably use MSW and lemongrass biomass, pyrolysis offers an environmentally sustainable alternative for reusing these byproducts. The anoxic decomposition of biomass is characterized by heat, a process of thermal degradation of biomass or material composed of organic and inorganic fractions, which saves time and space for waste management. Its products include biochar (solid fraction), bio-oil (liquid fraction), and noncondensable gases (gaseous fraction), whose yield proportions are a function of the raw material, the type of reactor used, and the operating conditions of the pyrolysis process.^11,12^

The thermochemical conversion process via pyrolysis is a technology for valuing biomass.^13^ This process involves the proper disposal of plant biomass and waste, such as sewage sludge, manure, plastics, paper, and rubber, among others, to produce biochar. This carbonaceous product can be used in various applications, including energy production, industry, and agriculture.^12^

With the development of carbon-based materials, such as activated carbon and carbon nanotubes, there has been a growing demand for alternative applications for such materials. Various studies on biochar began to be developed to improve the material’s physical-chemical characteristics. Such studies aim to enhance the applicability of biochar, primarily as a source of energy storage and conversion, and to design viable, sustainable applications based on its use as a reaction catalyst, a capacitor, and as nanotubes and composites for inorganic and biological applications.^14^

The agronomic effects of biochar are mainly determined by its physicochemical properties and how these interact with the substrate-plant system.^15^ Biochar has been associated with physical, chemical, and biological improvements in the soil, influencing plant development through different mechanisms: improvement of the physical structure of the substrate, with a possible elevation of porosity and aeration; greater water retention due to its highly porous structure; pH modulation, especially in acidic substrates, due to the presence of alkaline mineral components and potentially influencing nutrient dynamics through adsorption and desorption processes, mediated by surface functional groups and cation exchange capacity.^13,15–18^

In addition, biochar can act as a slow-release source of nutrients and as a buffer system against losses through leaching, contributing to greater efficiency in nutrient use by plants. These mechanisms occur simultaneously and depend directly on the raw material used and the pyrolysis conditions, which determine the chemical composition and structure of the material.^19,20^

Biochar has been extensively investigated as a substrate and soil conditioner due to its high surface area, chemical stability, and water- and nutrient-retention capacity. It can improve plant growth and nutritional efficiency.^21,22^ In seedling production systems, incorporating biochar into the substrate can modify physical and chemical properties of the growing medium, such as porosity, pH, cation exchange capacity, and nutrient availability, favoring root development and initial plant growth.^23,24^

The use of biochar in mixtures with substrates is associated with the association of pyrogenic carbon present in its fragments with humified organic matter, as well as with the contribution of nutrients to the composition of the mixture.^25^ Among possible applications, using biochar in soil has promising potential as a recalcitrant carbon source and as a soil conditioner/improver.^14^

The use of substrate cultivation systems is becoming a growing, sustainable alternative to preserve the soil, as substrates are composed of mixtures of organic materials (rice husks, humus, peat) and minerals (perlite and vermiculite), with formulations varying depending on management (hydroponic, semihydroponic) and cultivation. This material supports root fixation and helps retain water and nutrients for plants, although it may be inert from a chemical perspective.^26^ The feasibility of using biochar as a component of the substrate for eucalyptus seedlings was verified by Peter et al. at a dose of 7.5 wt %, with greater accumulation of aerial fresh mass than in plants grown without biochar application.^15^

Despite the growing interest in the use of biochar as a conditioner for agricultural substrates, comparative studies evaluating biochars produced from different waste sources, especially municipal solid waste and residual biomass from aromatic plants, are still limited. Most previously published work focuses on biochars obtained from a single raw material, with little emphasis on how differences in the physicochemical properties of these materials simultaneously influence nutrient availability and the initial development of seedlings of species with distinct physiological requirements. This knowledge gap is particularly relevant in seedling production systems, where substrate composition directly influences plant establishment, nutrient uptake, and initial growth.

Furthermore, the agronomic potential of biochars derived from municipal solid waste remains underexplored compared to conventional lignocellulosic wastes, despite their significant relevance to sustainable waste management strategies and the circular economy. In this context, the present study aimed to comparatively evaluate the effects of biochars produced from municipal solid waste and lemongrass residual biomass on the production of basil (*Ocimum basilicum* L.) and eucalyptus (*Eucalyptus grandis* W. Hill) seedlings, considering their physicochemical properties, nutrient dynamics, and influence on plant biometric development. By integrating structural characterization via FTIR and XRD with nutritional and plant growth responses, this study contributes to a broader understanding of how biochar origin and composition affect its agronomic performance as a substrate amendment. Therefore, rather than assessing the general efficacy of biochar as a plant-growth-promoting amendment, this study focused on comparing the responses of two plant species to biochars produced from contrasting waste sources under identical greenhouse conditions.

## 2 Materials and Methods

### 2.1 Biochar Obtainment

The biochar used was derived from municipal solid waste (MSW), sampled according to the procedures described in the ASTM D5231–92 and ABNT NBR 10007/2004 standards, and from residual biomass obtained during the extraction of lemongrass essential oil (*C. citratus*). The experiment was conducted using MSW biochar (BMSW) and lemongrass biochar (BCL), both obtained by slow pyrolysis in a screw reactor with a residence time of 30 min and temperatures of 450 °C for BMSW and 350 °C for BCL. After obtaining the biochar, the material was ground in a sieve with a 2.0 mm mesh opening (9 mesh/Tyler).

The different pyrolysis temperatures were selected based on the specific characteristics of each feedstock and on previous process optimization studies conducted by Bisi,^27^ which evaluated the influence of pyrolysis conditions on the yield, physicochemical properties, and agronomic suitability of the biochar. However, it is recognized that this variation can influence physicochemical properties, such as pH, ash content, aromaticity, and mineral and structural composition, and should be considered in the comparative interpretation of the results. The biochars used in this study correspond to the same batches previously characterized by Bisi^27^ and both demonstrated favorable agronomic characteristics for use as substrate conditioners.

In terms of pH, both biochars exhibit alkaline character (BMSW: 9.58 and BCL: 8.83) and calcium contents of 59.4 g·kg^–1^ for BMSW and 9.2 g·kg^–1^ for BCL, indicating possible potential for use as a soil amendment.^28^ Notably, their porosity and surface area favor greater physical interaction with the substrate matrix and the microorganisms present; BET analysis revealed that the surface area of BMSW is 7.56 m^2^·g^–1^ and that of BCL is 4.92 m^2^·g^–1^. Chemical analysis demonstrated the presence of macronutrients essential for plant growth (BMSW: *N* = 18.8 g·kg^–1^, *P* = 8.3 g·kg^–1^, *K* = 14.5 g·kg^–1^, Mg = 3.4 g·kg^–1^, and BCL: *N* = 13.3 g·kg^–1^, *P* = 4.0 g·kg^–1^, *K* = 38.0 g·kg^–1^, Mg = 5.7 g·kg^–1^), in addition to the ability to raise the substrate pH to adequate levels and allow the supply of calcium, as mentioned earlier, mitigating the potential acidification effects possibly caused by the use of nutrient solution.^27^

In the analysis of heavy metals, no critical levels were observed in any of the biochars evaluated, since all the determined values remained below the maximum limits established by CONAMA Resolution No. 375/2006. For BCL, the levels obtained were: Cd: 0.23 mg·kg^–1^; Pb: 3.05 mg·kg^–1^; Cu: 41.89 mg·kg^–1^; Cr: 3.92 mg·kg^–1^; Mo: 1.40 mg·kg^–1^; Ni: 2.35 mg·kg^–1^ and Zn: 85.10 mg·kg^–1^. The elements As and Hg had concentrations below the detection limit of the analytical method. For BMSW, the levels determined were: Cd: 0.74 mg·kg^–1^; Pb: 20.20 mg·kg^–1^; Cu: 38.67 mg·kg^–1^; Cr: 36.12 mg·kg^–1^; Mo: 10.53 mg·kg^–1^; Ni: 33.67 mg·kg^–1^ and Zn: 587.60 mg·kg^–1^. Similarly, As and Hg also remained below the method’s detection limit.^27^

The levels of potentially toxic metals remained below the limits established by CONAMA Resolution No. 375/2006, indicating compliance with regulatory standards for agricultural use under the evaluated conditions. However, caution is recommended regarding the continuous application of these materials, especially in relation to BMSW, and long-term assessments related to stability and the potential accumulation of contaminants in the environment are important.

The characterization of biochars by Fourier transform infrared spectroscopy (FTIR) was performed using previously sieved samples (<200 mesh), mixed with spectroscopic grade KBr and pressed into pellet form for reading in an FTIR spectrometer, in the range of 4,300–400 cm^–1^.

The analysis aimed to identify the main functional groups present on the surface of the biochars, especially those related to nutrient retention and exchange, such as hydroxyls, carbonyls, carboxyls, and nitrogen compounds. These groups can directly influence ion adsorption, interaction with the nutrient solution, and the behavior of the biochar as a substrate conditioner.

X-ray diffraction (XRD) was used to identify crystalline mineral phases and evaluate the structural organization of the biochars. The previously dried and sieved samples (<200 mesh) were analyzed in a diffractometer with a copper anode, using a scan from 5° to 90° for 2 h, with a step of 0.05°·s^–1^ and an integration time of 4 s.

The technique allowed us to evaluate the predominance of crystalline and amorphous structures, as well as to identify inorganic minerals, such as quartz and calcium carbonate, associated with changes in pH, nutrient availability, and structural stability of the material in the substrate. Thus, the combined use of FTIR and XRD techniques enabled a more comprehensive characterization of biochars and a deeper understanding of their functional behavior in agricultural systems.

While FTIR provided information on surface functional groups related to chemical reactivity and ion exchange, XRD complemented this analysis by identifying mineral phases that can act as nutrient sources or influence the pH of the substrate. Together, these techniques are fundamental not only for the structural characterization of the material, but also for inferring possible interaction mechanisms between biochar, nutrients and the root system of plants.^27^

Chemical analysis of the substrate after incorporation of the biochars was not performed, since the main objective of the study was to evaluate the direct effect of the intrinsic characteristics of the previously characterized biochars on plant development. As cultivation was conducted in a commercial substrate considered chemically stable and with standardized supplementation of Hoagland and Arnon nutrient solution, the aim was to minimize external interferences and prioritize the comparative evaluation of the agronomic response associated with the origin and composition of the biochars.

### 2.2 Experiment Implementation

Basil seeds (*O. basilicum* L.) of the variety ″Fine French Basil″ from the company Feltrin Sementes (Farroupilha, Brazil) and white eucalyptus (*E. grandis* W.Hill) from the company Sementes Caiçara (Brejo Alegre, Brazil) were used in the experiments.

Different proportions of biochar were incorporated into the commercial substrate in order to evaluate its effects on the growth and nutrition of basil and eucalyptus seedlings. The treatments consisted of mixtures of the commercial substrate Carolina Soil (Santa Cruz do Sul, Brazil) with different proportions of biochar. The commercial substrate is composed of vermiculite, expanded perlite, sphagnum peat, and toasted rice husk. According to the manufacturer, the material has a pH of 5.5 ± 0.5 and an electrical conductivity of 0.7 ± 0.3 mS·cm^–1^.

The biochar concentrations were defined based on previous studies that demonstrate that proportions between 5 and 10 wt % can promote improvements in the physical and chemical properties of the substrate without causing negative effects on plant development,.^8,2^

The experiment was conducted in a completely randomized design, with three replicates per treatment, each replicate consisting of an experimental unit composed of ten plants. Individual evaluations were used to calculate the average per replicate, and these were considered in the statistical analyses, totaling three experimental units per treatment. In total, there were seven treatments: two biochar sources (BMSW and BCL) applied at three concentrations (5.0, 7.5, and 10.0 wt %) and a control treatment without biochar. The commercial substrate was previously homogenized with the BMSW and BCL biochars in the proportions defined for each treatment. Subsequently, the mixtures were packaged in conical plastic containers with a volume of 30 cm^3^, considering the total mass of substrate in each container, according to the coding and details compiled in Table 1.

**Table 1:** Coding and Description of the Treatments Used in the Experiment with Basil and Eucalyptus^a^

codification	treatment
T0	Substrate only (control)
T1	Substrate and 5.0 wt % BMSW
T2	Substrate and 7.5 wt % BMSW
T3	Substrate and 10.0 wt % BMSW
T4	Substrate and 5.0 wt % BCL
T5	Substrate and 7.5 wt % BCL
T6	Substrate and 10.0 wt % BCL

a Caption: BMSW: municipal solid waste biochar; BCL: lemongrass biomass biochar.

The basil plants were grown for 60 days, between December 2022 and February 2023, and the eucalyptus plants for 90 days, between December 2022 and March 2023, in greenhouse conditions with irrigation via microsprinklers. After 15 days of seed germination, the plants received, individually, 3.0 mL of nutrient solution prepared according to Hoagland and Arnon^28^ twice a week until the end of the experiment. During the experimental period, the average temperature was 22.7 °C, with a maximum of 33.4 °C and a minimum of 8.2 °C, and the average relative humidity was 71.1%.

### 2.3 Assessment of Plant Parameters

After the experiment, the basil and eucalyptus plants were evaluated for the length of the aerial part using a digital caliper, and root volume was determined by immersing the roots in a graduated cylinder containing a known volume of water, with the difference observed as the root volume. For dry-mass determination, plant material was dried in a forced-air circulation kiln at 60 °C for 24 h, and the dry mass was then determined.

The nutritional contents of phosphorus, potassium, calcium, and magnesium in plant tissue were evaluated in both cultures; boron was evaluated in the basil crop, and copper content in eucalyptus plants was evaluated. Assessments of nutritional contents in plant material were carried out according to methods described by Malavolta.^29^

As in the experiment, the plants were grown in an inert substrate containing biochar at different concentrations; nutritional analysis of the substrate was not performed, as the biochar was characterized separately before the experiment.

### 2.4 Statistical Analysis

For statistical analysis, treatments containing biochar were evaluated in a 2 × 3 factorial arrangement (biochar type × concentration), with the control included in the ANOVA for comparison. The data were evaluated for homoscedasticity (Levene’s test) and normality of residuals (Shapiro-Wilk test), being subjected to Analysis of Variance (ANOVA). Subsequently, the means were compared using the Tukey test at a 5% probability of error (α = 0.05) using the AgroEstat software (Brazil).

### 2.5 Use of Artificial Intelligence

Artificial intelligence tools were used exclusively to support the linguistic review (grammatical, spelling, and style corrections) of the manuscript, as well as auxiliary tools for searching for scientific articles. The identification, selection, and interpretation of the scientific articles cited were conducted by the authors. All scientific decisions, analyses, results, and conclusions presented in this work are the sole responsibility of the authors.

## 3 Results and Discussion

### 3.1 Analysis of FTIR Data Obtained from Biochars

Fourier transform infrared (FTIR) spectroscopy analysis was employed to identify the main functional groups present in lemongrass (BCL) and municipal solid waste (BMSW) biochars, allowing inferences about their surface chemical properties and potential agronomic applications. FTIR is widely used in the characterization of biochars because it provides direct information about functional groups responsible for the reactivity, stability, and adsorption capacity of these materials.^30,31^

Figure 1 shows the FTIR spectra of the biochar samples, highlighting the main absorption bands associated with the functional groups present on the surface of the material.

**Figure 1:**
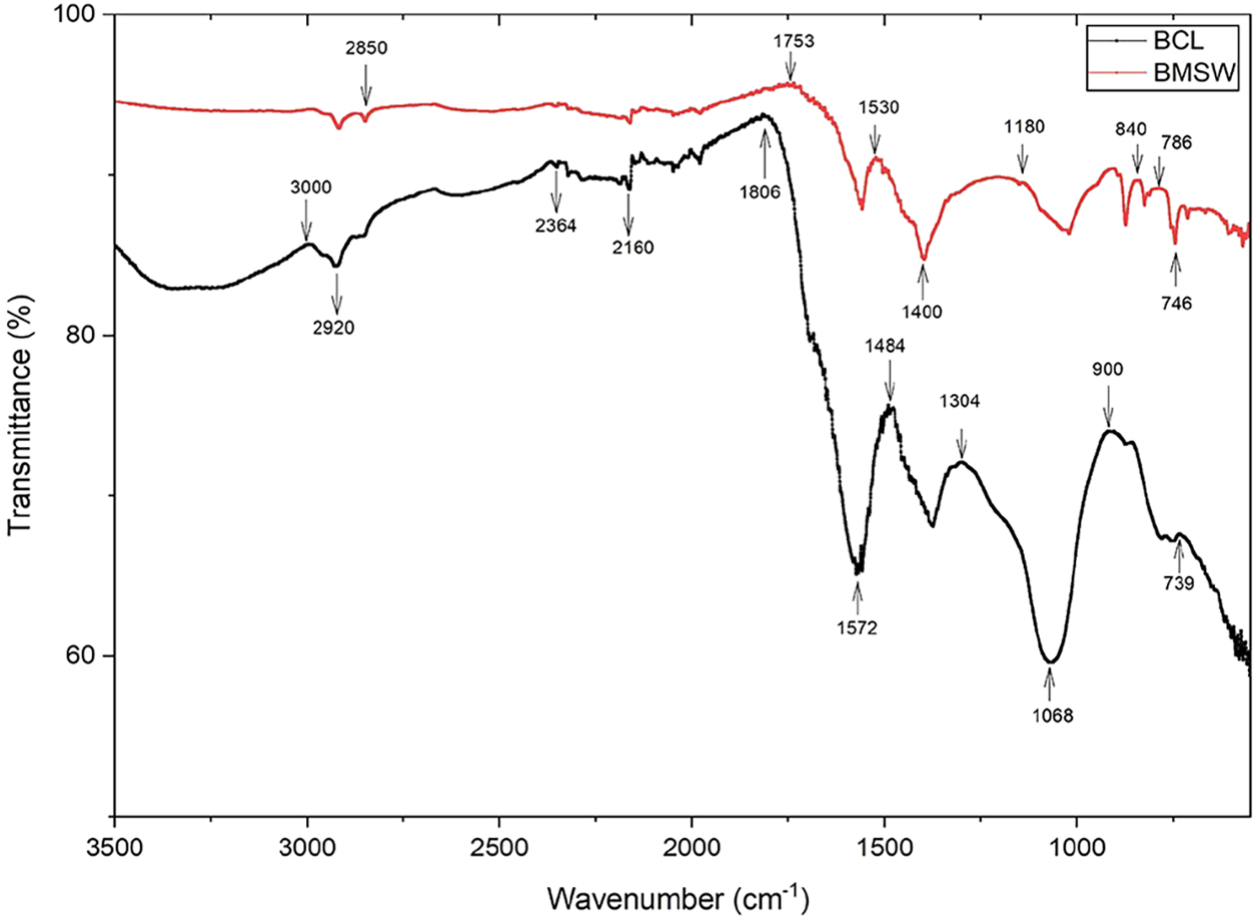
FTIR spectra of lemongrass (BCL) and municipal solid waste (BMSW) biochar samples.

Table 2 shows the main FTIR spectral bands associated with the BCL and BMSW biochars and their agronomic potential.

**Table 2:** Main FTIR Bands Identified in the Studied Biochars and Their Potential Agronomic Relevance

wavenumber (cm^–1^)	attribution	biochar	potential agronomic relevance
2920–3000	aliphatic C–H	BCL/BMSW	Residual organic structures^30,32^
2364–2160	C≡C/C≡N	BCL	Surface reactivity^31,33^
1806–1753	C=O (carbonyl)	BCL/BMSW	Potential nutrient retention and ion exchange^33,34^
1572–1530	aromatic C=C	BCL/BMSW	Structural stability and persistence in the substrate^30,31,35–37^
1484–1304	phenolic groups	BCL	Chemical interaction with nutrients^30,39^
1068–1180	C–O/Si–O	BCL/BMSW	Nutrient adsorption and mineral interactions^30,39^
840–739	aromatic C–H/silicates	BCL/BMSW	Structural and mineral contribution^30,39^

Although these attributes suggest possible interactions with nutrient dynamics and substrate chemistry, the present study did not directly evaluate nutrient retention mechanisms or substrate chemical properties following biochar incorporation.

In general, the BCL spectrum showed more intense and well-defined bands, indicating a greater predominance of aromatic organic structures and oxygenated functional groups, while the BMSW showed a more attenuated spectrum, with a greater contribution of mineral phases and less organic complexity, a behavior frequently observed in biochars derived from heterogeneous residues.^32^

For both samples (BCL and BMSW), the bands observed in the 2920–3000 cm^–1^ region were attributed to the stretching of C–H bonds of aliphatic groups (−CH_2_ and −CH_3_), indicating the residual presence of organic chains from partially degraded cellulose and hemicellulose. The reduction of these bands after pyrolysis has been associated with the thermal decomposition of biomass and the formation of more condensed aromatic structures.^30,32^

In the BCL, the bands at 2364 and 2160 cm^–1^ may be related to C≡C and C≡N bonds, frequently reported in biochars because of structural rearrangements during pyrolysis.^31,33^ The presence of a band around 1806 cm^–1^ indicates carbonyl groups (C=O), including ketones, aldehydes, and carboxylic acids. These groups have been associated in literature with possible interactions involving nutrient retention and ion exchange properties in carbonaceous materials.^33,34^

The most intense band in the BCL, at 1572 cm^–1^, can be attributed to the stretching of aromatic C=C bonds, suggesting a higher degree of aromaticity and structural condensation. This behavior is frequently related to the greater chemical stability of the material and the persistence of aromatic carbonaceous structures after pyrolysis.^30,31,35–37^ In the region between 1484 and 1304 cm^–1^, bands associated with the deformation of aliphatic groups and the presence of phenolic compounds derived from lignin were observed. The intense band at 1068 cm^–1^ can be attributed to the C–O stretching of alcohols, phenols, and ethers, and may also indicate contributions from Si–O bonds associated with the residual mineral fraction.^30^ The bands observed at 900 and 739 cm^–1^ correspond to the out-of-plane deformation of aromatic C–H bonds, suggesting the presence of polycondensed aromatic structures typical of carbonized materials.^31,35,37,38^

For BMSW, the spectrum showed distinct characteristics. The band at 2850 cm^–1^ was attributed to aliphatic C–H stretching, but with lower intensity, indicating a lower predominance of organic structures compared to BCL.^30^ The band at 1753 cm^–1^ was associated with carbonyl groups (C=O), including carboxylic acids and esters. Although present, its lower intensity suggests a lower relative abundance of oxygenated groups when compared to BCL.^34^ The band at 1530 cm^–1^ indicates the presence of aromatic structures (C=C), but also with lower intensity, suggesting a lower degree of aromatic condensation compared to lignocellulosic biochar.^30^

The band at 1400 cm^–1^ can be attributed to carbonates (CO_3_^2–^) and carboxylate groups, indicating a greater mineral contribution. This behavior is characteristic of biochars derived from urban waste, which generally have a higher ash and inorganic compound content.^30,39^ The band at 1180 cm^–1^ is related to C–O and Si–O stretching, simultaneously indicating residual organic matter and silicates. The bands at 840 cm^–1^, 786 cm^–1^, and 746 cm^–1^ reinforce the presence of mineral structures, especially Si–O–Si bonds associated with inorganic fractions present in urban solid waste.^30,39^

Comparatively, BCL showed a higher relative intensity of oxygenated functional groups and aromatic structures, while BMSW showed a greater mineral contribution and lower organic complexity. These differences may be related to the characteristics of the raw materials and the pyrolysis conditions employed, factors that are recognized as important in defining the physicochemical properties of biochars.^30,33^ The oxygenated groups identified for BCL and BMSW, such as carbonyls and hydroxyls, as well as nitrogenous groups, have been associated in the literature with possible interactions involving adsorption, ion exchange, and chemical modifications in the substrate.^40,41^ However, although the identification of these groups by FTIR indicates potential chemical reactivity, the techniques employed do not allow for the direct elucidation of the mechanisms involved in the interactions between biochar, nutrients, and plants. Therefore, the interpretations presented should be considered as inferences based on the specialized literature and the experimental results obtained.

The differences observed in the FTIR spectra may help interpret, at least partially, the contrasting agronomic responses observed in the basil and eucalyptus seedlings. The greater abundance of oxygenated functional groups identified in BCL—particularly carbonyl, phenolic, and C–O bond-containing groups—may be associated with a higher nutrient retention and exchange capacity, which could contribute to the improved root development and greater biomass accumulation observed in the basil seedlings. Conversely, the higher mineral contribution observed in BMSW, along with the presence of carbonate-associated bands, may be related to the greater calcium accumulation and improved shoot growth observed in the eucalyptus seedlings. However, these interpretations should be viewed with caution, as the physicochemical properties of the substrates following biochar incorporation were not measured, and a direct causal relationship cannot be established.

### 3.2 Analysis of Data Obtained by XRD in Biochars

X-ray diffraction (XRD) was used to evaluate the crystalline structure and degree of organization of BCL and BMSW, allowing the identification of mineral phases and inferring the degree of carbon amorphization. This technique is widely used in the characterization of biochars because it allows the distinction between inorganic crystalline phases and amorphous or partially ordered carbonaceous structures.^33,42^

Figure 2 shows the diffractograms of the samples, highlighting striking differences between the materials. In general, BCL showed a pattern dominated by a broad and diffuse band, while BMSW exhibited more defined and intense peaks, characteristic of mineral crystalline phases.

**Figure 2:**
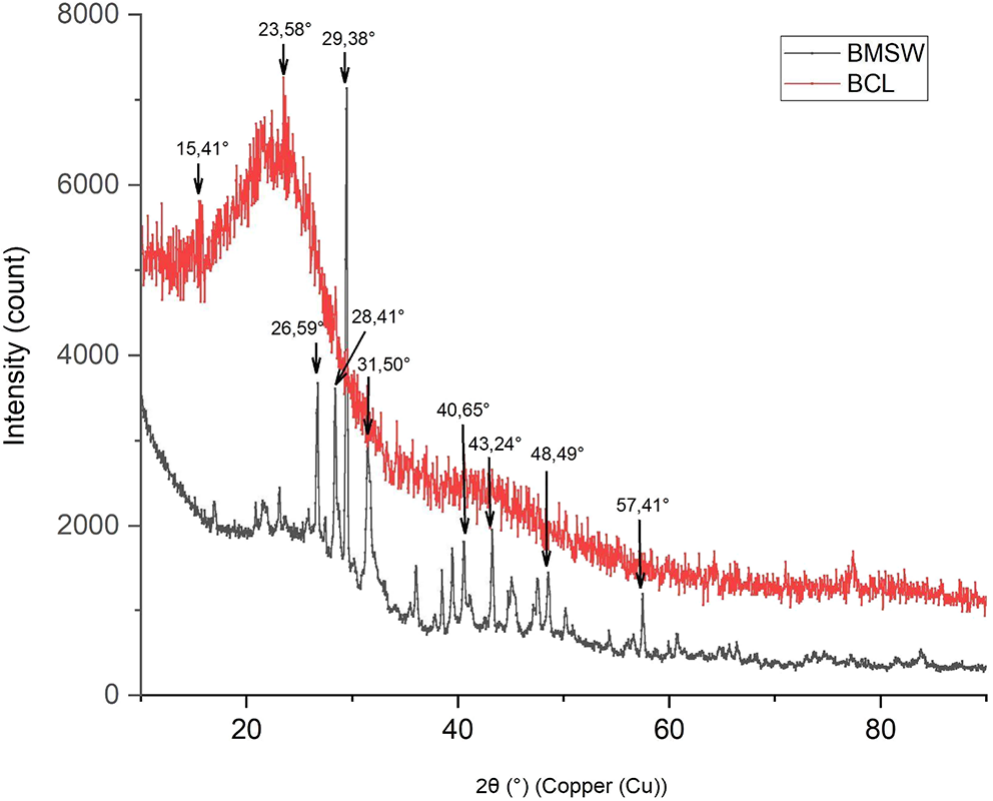
X-ray diffractograms (XRD) of lemongrass biochars (BCL) and municipal solid waste biochars (BMSW).

The diffractograms were obtained using Cu–Kα radiation, with 2θ scanning between 5° and 90°. The diffraction pattern of BCL showed a predominantly amorphous character, with a low-intensity halo centered between 10° and 30° (2θ). BMSW, on the other hand, showed more defined peaks between 20° and 60° (2θ), in addition to signals distributed throughout the diffractogram, suggesting a greater contribution from mineral phases remaining from the pyrolysis process.^43^

In BCL, a broad band centered between 23° and 26° (2θ) was observed, with a maximum at approximately 23.58° (2θ), a characteristic frequently associated with amorphous or weakly organized carbonaceous structures, related to turbostratic carbon. This pattern suggests a low degree of crystallinity and greater structural disorder, a behavior commonly reported for lignocellulosic biochars produced at moderate temperatures.^33,44^

The BMSW presented a diffractogram with multiple, more defined peaks, indicating a greater relative contribution of crystalline phases. The intense peak at 29.38° (2θ) can be attributed to calcite (CaCO_3_), a phase frequently identified in biochars derived from urban waste due to its higher ash content and the presence of carbonate materials.^45–47^

The presence of a shoulder around 43.24° (2θ) in the BMSW may be related to the aromatic carbon plane, suggesting a certain degree of structural organization of the carbonaceous layers, albeit limited. This behavior is consistent with the results obtained by FTIR, which indicated the presence of condensed aromatic structures.^30,42^

Additionally, the peaks observed at 26.59° and 28.41° (2θ) may be associated with the presence of quartz (SiO_2_) and silicates, while signals at 31.50°, 40.65°, 43.24° and 57.41° (2θ) suggest the occurrence of other mineral phases, including silicates and possible metallic oxides, reflecting the heterogeneity characteristic of municipal solid waste.^44,46–48^ Comparatively, BMSW showed a lower relative contribution of the amorphous phase and a greater predominance of crystalline structures, a behavior frequently associated with the higher ash content of this type of material.^45^

In general, BCL showed a predominantly amorphous structure, with evidence of limited aromatic organization, while BMSW showed a greater mineral contribution and a greater relative presence of crystalline phases, including carbonates and silicates.^21,44^ These differences may be related to both the characteristics of the raw materials and the pyrolysis conditions employed, factors that are recognized as important in defining the structural properties of biochars.^30,42,43,48^

The presence of mineral phases such as quartz (SiO_2_) and calcium carbonate (CaCO_3_) may be associated with changes in the chemical properties of the substrate, especially in relation to pH and mineral availability. In the case of BMSW, the presence of carbonates may be related to the higher calcium content observed in plant tissue in some treatments. In addition, the mineral fraction may influence physical characteristics of the material, such as density and porosity, although these parameters were not directly evaluated in this study.^21,49^

However, it should be noted that the XRD technique allows the identification of predominantly crystalline phases and general structural characteristics of the materials, not enabling the direct determination of the mechanisms involved in the interactions between biochar, nutrients and plants. Thus, the interpretations presented should be considered as inferences based on the specialized literature and the known properties of biochars.

### 3.3 Evaluation of the Effects of Applying Biochars to Basil Seedlings

The results of applying biochars on the biometric parameters of the seedlings are shown in Table 3.

**Table 3:** Biometric Parameters of Basil (*O. basilicum* L.) Seedlings Grown with Increasing Concentrations of BMSW and BCL Incorporated into the Substrate^a^

	BMSW	BCL	BMSW	BCL	BMSW	BCL	BMSW	BCL	BMSW	BCL
	Aerial part length		Root length		Aerial dry mass		Dry root mass		Root volume	
concentration	(cm)		(cm)		(g)		(g)		(mL)	
Zero	24.70 Aa	24.70 Ab	9.98 Aa	9.98 Aa	0.39 Aa	0.39 Aa	0.11 Aa	0.11 Aa	2.28 Aa	2.28 Aa
5.0 wt %	23.03 Ba	31.50 Aa	7.83 Ab	7.84 Ab	0.30 Ba	0.58 Aa	0.07 Ba	0.12 Aa	1.46 Bb	2.83 Aa
7.5 wt %	24.27 Ba	33.68 Aa	7.94 Ab	8.19 Ab	0.33 Ba	0.56 Aa	0.08 Ba	0.10 Aa	1.76 Bab	2.38 Aa
10.0 wt %	23.01 Ba	33.43 Aa	7.97 Ab	7.52 Ab	0.34 Ba	0.53 Aa	0.09 Ba	0.11 Aa	2.28 Ba	2.33 Aa
SD	1.06		0.31		0.04		0.02		0.30	
CV (%)	3.87		3.73		8.71		18.81		13.66	

a SD: standard deviation. CV: coefficient of variation. BCL: lemongrass biochar; BMSW: municipal solid waste biochar. Means followed by the same lowercase letter in column (concentration) and by the same uppercase letter (biochar type) did not differ by Tukey’s test at a 5% error probability (*p* ≤ 0.05).

Root volume, however, was greater in treatments containing BCL compared to treatments with BMSW, regardless of biochar concentration. In general, BCL showed better performance for most of the biometric parameters evaluated. Considering shoot length and shoot and root dry mass, greater biomass accumulation was observed in treatments containing BCL compared to those containing BMSW. In contrast, Soares did not observe reductions in shoot length, root length, or dry biomass accumulation with increasing concentrations of eucalyptus biochar in substrates used for *Sapindus saponaria* L. seedlings, differing from the behavior observed in the present study for treatments with BCL.^50^

These differences may be related to variations in the physicochemical properties of biochars, since the composition of the raw material and the pyrolysis temperature are known factors that influence characteristics such as pH, mineral composition, porosity, and nutrient retention capacity, and may affect plant development and nutrient dynamics in the substrate.^21,23,24^

The biometric responses observed in some treatments may also be associated with physical modifications in the substrate promoted by the incorporation of biochar, including possible changes in porosity, aeration, and water retention. In addition, the porous structure and surface characteristics of biochars have been associated with interactions involving nutrient retention and availability in growing substrates.^51,52^ These factors may have contributed, at least partially, to the differences observed in root development and biomass accumulation between treatments.

In addition, differences in the surface characteristics and porosity of biochars may have influenced nutrient dynamics in the substrate. Materials with a larger surface area are often associated with a greater capacity for water and nutrient retention, which can affect the availability of these elements over time.^21,51^ Although these properties were not directly measured in the present study, such interactions can help in the interpretation of the variations observed in biometric parameters, especially in root volume and dry mass of basil plants grown in substrates containing biochar.

The macro and micronutrient contents determined in the plant tissue of basil grown in substrates containing BMSW and BCL are presented in Table 3 for P, K, Ca, Mg, and B. No significant differences were observed for the N content, with average values of 8.14 g·kg^–1^ for BMSW and 7.65 g·kg^–1^ for BCL. Nitrogen is an essential nutrient for vegetative development and, although no statistical differences were observed, the variations in plant growth between treatments may be associated with differences in nutrient availability and in the physicochemical characteristics of the substrates containing biochar.

Basil plants showed different patterns of nutrient accumulation depending on the biochar source. In general, plant tissues showed higher levels of P, Ca, and Mg in treatments containing BMSW, while higher levels of K and B were observed in treatments with BCL. Similar effects associated with the type of biochar and nutrient uptake by plants were also reported by Castro-Herrera.^53^ This behavior may also be related to the original chemical composition of the biochars, since BCL showed a higher K content, while BMSW showed higher concentrations of Ca. Thus, part of the differences observed in nutrient levels in plant tissue may reflect the contribution of biochars as supplementary sources of these elements in the substrate.

The incorporation of biochar into the substrate can also influence nutrient dynamics through processes associated with adsorption, retention on porous surfaces, and changes in the chemical characteristics of the substrate, potentially affecting nutrient availability to plants.^21^ It is worth noting that all treatments, including the control, received Hoagland nutrient solution throughout the experiment, ensuring similar baseline nutritional conditions. However, this approach may have reduced differences associated exclusively with the supply of nutrients by the biochars, making it difficult to separate the nutritional effects from other physicochemical influences of the materials on plant growth. This effect can be observed in Table 4.

**Table 4:** Nutritional Contents in the Plant Material of Basil Seedlings Cultivated for 60 Days with Increasing Concentrations of BMSW and BCL in a Commercial Substrate^a^

	BMSW	BCL	BMSW	BCL	BMSW	BCL	BMSW	BCL	BMSW	BCL	
concentration	P (g·kg^–1^)		K (g·kg^–1^)		Ca (g·kg^–1^)		Mg (g·kg^–1^)		B (mg·kg^–1^)		
Zero	38.5 Ac	38.5 Ab	10.2Ac	10.2Ac	17.3Ad	17.3 Aa	7.3 Aa	7.3 Aa	10.1Ad	10.1 Ab	
5.0 wt %	39.3 Ab	37.2 Bd	13.8 Aab	13.5 Ab	21.9 Ab	14.6 Bb	7.3 Aa	5.5 Bc	11.6 Bc	16.3 Aa	
7.5 wt %	39.9 Aa	39.9 Aa	14.3 Ba	15.3 Aa	19.6Ac	14.6 Bb	6.0 Ac	5.8 Bb	15.5 Bb	16.7 Aa	
10.0 wt %	38.5Ac	37.8 Bc	13.3 Bb	15.0 Aa	23.2 Aa	14.9 Bb	6.6 Ab	5.7 Bb	18.4 Aa	16.5 Ba	
SD	0.1		0.3		0.3		0.1		0.5		
CV (%)	0.4		1.9		1.6		0.6		3.4		

a SD: standard deviation. CV: coefficient of variation. BCL: lemongrass biochar; BMSW: municipal solid waste biochar. Means followed by the same lowercase letter in column (concentration) and by the same uppercase letter (biochar type) did not differ by Tukey’s test at a 5% error probability (*p* ≤ 0.05).

When evaluating the phosphorus (P) content, it was found that the concentration of 7.5 wt % resulted in the highest levels of this nutrient in the plant tissue, regardless of the type of biochar. For calcium (Ca), the highest concentrations in the plant material were observed in the treatments containing 10.0 wt % of BMSW. The accumulation of magnesium (Mg) showed statistical differences between the treatments with BMSW, with the concentration of 5.0 wt % showing the highest Mg content without differing from the control treatment. In general, plants grown with BMSW showed higher concentrations of P compared to those grown with BCL.

Although BCL had a higher total Mg content in its composition, the higher concentration of this nutrient observed in the plant tissues grown with BMSW suggests that the availability of the element in the substrate may have differed between treatments. This behavior may be related to differences in the physicochemical characteristics of the biochars, including possible effects associated with nutrient retention, release dynamics, or ionic interactions in the substrate, such as competition between Ca^2+^ and Mg^2+^. Mg also participates in P transport and energy transfer processes in plants.^29^ However, these interactions were not directly evaluated in the present study.

Another relevant observation refers to P levels in plant tissue. Although plants grown with BMSW showed higher P concentrations compared to those grown with BCL, this increase was not accompanied by greater plant growth. This behavior may be associated with a possible nutritional dilution effect, in which plants with greater biomass accumulation have lower relative concentrations of certain nutrients due to the distribution of these elements in a greater amount of dry matter. However, the effects of nutritional dilution and nutrient mass balance were not directly evaluated in this study. Considering that phosphorus plays an essential role in ATP synthesis, nucleic acid formation, and cell division processes, variations in the concentration of this nutrient may be associated with differences in biomass production and nutrient distribution in plant tissues.^54^

The analysis of potassium (K) content in plant tissue indicated that the application of BCL above 7.5 wt % was associated with greater accumulation of this nutrient in plants. This result may be related to the higher potassium content originally present in BCL compared to BMSW. Regarding the micronutrient boron (B), higher concentrations were observed in plant material grown with 5.0 wt % of BCL and 10.0 wt % of BMSW. Similar to the behavior observed for Mg, these differences may be associated with the original composition of the biochars and possible variations in nutrient availability in the substrate.

Different patterns of nutrient accumulation between BMSW and BCL were also reported by Castro-Herrera,^55^ who highlighted that the nutritional differences associated with biochar application can vary according to the raw material composition and the material production conditions.

### 3.4 Evaluation of the Effects of Biochar Application on Eucalyptus Seedlings

As with basil, all treatments, including the control, received a nutrient solution of Hoagland’s Solution, aiming to ensure similar nutritional conditions; however, this practice may have attenuated the differences related to the direct supply of nutrients by the biochars. The biometric parameters evaluated in eucalyptus seedlings grown in substrate containing both biochars are presented in Table 5.

**Table 5:** Biometric Parameters of Eucalyptus (*E. grandis*) Seedlings Grown with Increasing Concentrations of BMSW and BCL Incorporated into a Commercial Substrate^a^

	BMSW	BCL	BMSW	BCL	BMSW	BCL	BMSW	BCL	BMSW	BCL
	Aerial part length		Root length		Aerial dry mass		Dry root mass		Root volume	
concentration	(cm)		(cm)		(g)		(g)		(mL)	
Zero	18.41 Ab	18.41 Aa	8.86 Aa	8.86 Aa	0.49 Aa	0.49 Aa	0.13 Aa	0.13 Aa	1.19 Aa	1.19 Aa
5.0 wt %	23.78 Aa	10.96 Bb	8.24 Aa	8.43 Aa	0.54 Aa	0.25 Bb	0.13 Aa	0.06 Bb	1.07 Aa	0.97 Aa
7.5 wt %	21.79 Aa	11.72 Bb	8.51 Aa	8.88 Aa	0.50 Aa	0.22 Bb	0.24 Aa	0.06 Bb	1.08 Aa	1.16 Aa
10.0 wt %	18.74 Ab	12.75 Bb	9.12 Aa	8.74 Aa	0.48 Aa	0.28 Bb	0.12 Aa	0.08 Bb	1.23 Aa	0.99 Aa
SD	1.12		0.37		0.08		0.07		0.15	
CV (%)	6.58		4.24		20.10		55.56		13.29	

a SD: standard deviation. CV: coefficient of variation. BCL: lemongrass biochar; BMSW: municipal solid waste biochar. Means followed by the same lowercase letter in column (concentration) and by the same uppercase letter (biochar type) did not differ by Tukey’s test at a 5% error probability (*p* ≤ 0.05).

In eucalyptus seedlings, greater shoot length was observed in treatments containing BMSW at concentrations of 5.0 wt % and 7.5 wt %, with BMSW being superior to BCL at all concentrations evaluated. In contrast, root length and root volume did not differ significantly from the control treatment, regardless of the source or concentration of biochar applied.

Regarding shoot and root dry mass, treatments containing BMSW showed similar values to those observed in the control, suggesting that the incorporation of this biochar did not negatively affect the initial development of eucalyptus seedlings. In addition, BMSW generally resulted in higher biomass values compared to BCL, regardless of the concentration used. Similar results were reported by Maia,^25^ who observed greater shoot growth and possible increase shoot and root dry mass in *Tachigali vulgaris* seedlings grown with eucalyptus biochar compared to plants grown without biochar.

In general, the composition of the biochars may have influenced the nutritional supply and absorption of nutrients by the plants at different concentrations. Plants grown with BCL generally showed higher levels of P, K, and Cu compared to treatments with BMSW at all concentrations evaluated. As discussed earlier for basil, the higher P content observed in eucalyptus plants grown with BCL, even with less development and biomass production, may be associated with a possible nutritional dilution effect related to plant growth and nutrient distribution in the tissues.

The results of the assessment of the nutritional parameters of eucalyptus plant material grown in the substrate with different concentrations of BMSW and BCL are shown in Table 6.

**Table 6:** Nutritional Contents in Plant Material from Eucalyptus Seedlings Cultivated for 90 Days in a Substrate with Different Concentrations of BMSW and BCL^a^

	BMSW	BCL	BMSW	BCL	BMSW	BCL	BMSW	BCL	BMSW	BCL
concentration	P (g·kg^–1^)		K (g·kg^–1^)		Ca (g·kg^–1^)		Mg (g·kg^–1^)		Cu (mg·kg^–1^)	
Zero	2.4 Aa	2.4 Ac	14.8 Aab	14.8 Ac	4.5Ac	4.5 Aa	3.0 Ab	3.0 Aa	9.0 Ac	9.0 Ac
5.0 wt %	2.2 Bb	3.5 Ab	14.3 Bb	20.7 Ab	5.3 Ab	4.1 Bc	3.2 Aa	2.5 Bb	11.6 Bb	12.0 Ab
7.5 wt %	2.6 Ba	3.7 Aa	14.3 Bb	24.3 Aa	5.6 Aa	4.3 Bb	2.5 Ad	2.4 Bc	12.1 Ba	12.8 Aa
10.0 wt %	2.4 Bab	3.3 Ab	15.3 Ba	24.0 Aa	4.3 Ad	3.5 Bd	2.6 Ac	2.4 Bc	11.1 Bd	12.5 Aa
SD	0.1		0.4		0.1		0.0		0.1	
CV (%)	3.1		2.3		0.9		0.1		0.9	

a SD: standard deviation. CV: coefficient of variation. BCL: lemongrass biochar; BMSW: municipal solid waste biochar. Means followed by the same lowercase letter in column (concentration) and by the same uppercase letter (biochar type) did not differ by Tukey’s test at a 5% error probability (*p* ≤ 0.05).

For the elements K and Cu, the highest values were observed in the plant tissue in treatments containing between 7.5 and 10.0 wt % of BCL, higher than those observed at 5.0 wt % and in control. When BMSW was incorporated into the substrate, the concentration of 10.0 wt % showed the highest K content, while 7.5 wt % of BMSW showed the highest Cu content in the plant tissue. These differences may be related to the distinct chemical and mineral characteristics of biochars, which can influence the availability and absorption of these elements by plants.

The tendency for higher K content in the plant material of plants treated with BCL, compared to those treated with BMSW, may also be related to the greater accumulation of biomass observed in treatments containing BMSW, which may have contributed to lower relative concentrations of this nutrient in plant tissues. However, the effects of nutritional dilution were not directly evaluated in this study.

It should be noted that higher concentrations of certain nutrients do not always result in greater plant growth, especially in situations of possible nutritional imbalance. The relative excess of some elements can influence the absorption of other essential nutrients, affecting physiological processes and plant development.^29^

The differences observed in the nutritional content absorbed by eucalyptus seedlings may be related to the physicochemical characteristics of biochars, including the presence of oxygenated functional groups and mineral fractions in the material matrix. Previous studies suggest that these characteristics can influence interactions involving nutrient retention and availability in substrates containing biochar.^25^ The same authors highlight that different studies have been evaluating the use of biochar associated with mineral fertilizers with the aim of reducing the dependence on chemical fertilizers in eucalyptus crops.

The performance differences observed between BCL and BMSW may be associated with the distinct structural and mineral characteristics of the materials. BCL showed a greater predominance of oxygenated functional groups identified by FTIR, while BMSW showed a greater mineral contribution observed in XRD analyses, especially related to the presence of carbonate compounds. These differences may have indirectly influenced nutrient availability and plant behavior throughout cultivation.

In addition, it should be considered that differential responses between species may be associated with specific pH requirements and physiological particularities related to nutrient absorption and metabolism, influencing the efficiency of utilization of elements made available by biochar. It should be noted that, although the biochars were individually characterized, the physicochemical characterization of the substrates after incorporation was not performed, which limits the direct understanding of the effects on properties such as pH, electrical conductivity, and nutrient dynamics in the system.

In general, the results demonstrate that the effects of biochar vary according to the physicochemical properties of the materials and the plant species evaluated. Furthermore, they show that biochars produced from municipal solid waste and lemongrass biomass at different pyrolysis temperatures and processing conditions do not exhibit equivalent behavior when applied as substrate conditioners.

It is also worth noting that, in addition to statistical significance, the observed results have agronomic relevance, since improvements in root development and biomass accumulation may be associated with seedling quality and post-transplant establishment potential in plant production systems. In this context, while lemongrass biochar (BCL) favored the biometric development of basil seedlings, urban solid waste biochar (BMSW) showed better performance in the shoot growth of eucalyptus seedlings, evidencing specific responses depending on the plant species and the composition of the material.

Thus, the findings of this study contribute to the literature by showing that the selection of the biochar source should consider not only its availability, but also the interaction between its physicochemical properties and the physiological requirements of the crop of interest. It should be noted, however, that the results should be interpreted as preliminary evidence obtained under controlled greenhouse conditions, and further studies under field conditions are needed for validation on an agronomic scale.

In this context, the present study is mainly configured as a comparative evaluation of the performance of biochars from different origins in seedling production, not aiming to conclusively elucidate the mechanisms of action of biochar.

## 4 Conclusion

The results indicate that biochars produced from municipal solid waste (BMSW) and lemongrass biomass (BCL) were associated with differences in the initial development of the seedlings, with responses dependent on the plant species and the type of biochar used. BCL was associated with greater biometric development in basil seedlings, whereas BMSW was associated with greater shoot growth in eucalyptus seedlings. The observed differences may be related to the physicochemical properties of the biochars, including mineral composition, crystalline phases identified by XRD, and functional groups identified by FTIR. However, these techniques provide only general structural information, not allowing the direct determination of the mechanisms involved in plant responses. In addition, the absence of measurements regarding pH, electrical conductivity (EC), porosity, water retention, nutrient availability, and other physicochemical properties of the substrate mixtures following biochar incorporation limits more detailed interpretations of nutrient dynamics and plant responses. The observed responses were species-dependent and varied according to the characteristics of each biochar. However, the results should be considered preliminary, since the study was conducted under controlled greenhouse conditions and with nutritional supplementation via Hoagland solution, which may have attenuated differences related to the direct supply of nutrients by the biochars. In this way, the study contributes as a comparative evaluation of biochars from different origins for agronomic use, highlighting that the raw material and production conditions may influence their behavior. Additional studies under field conditions, with longer cultivation time and evaluation of the physicochemical properties of the substrate and environmental safety, are needed to broaden the understanding of its applicability. Therefore, the main contribution of this study is the comparative evaluation of two waste-derived biochars under identical cultivation conditions, providing evidence that plant responses are species-dependent and influenced by the characteristics of the biochar biomass.
